# Investigating neural mechanisms of change of cognitive behavioural therapy for chronic fatigue syndrome: a randomized controlled trial

**DOI:** 10.1186/s12888-015-0515-9

**Published:** 2015-07-03

**Authors:** Marieke E van Der Schaaf, Iris C Schmits, Megan Roerink, Dirk EM Geurts, Ivan Toni, Karin Roelofs, Floris P De Lange, Urs M Nater, Jos WM van der Meer, Hans Knoop

**Affiliations:** 1 Radboud University Medical Center, Expert Centre for Chronic Fatigue, Nijmegen, The Netherlands; 2 Donders Institute for Brain, Cognition, and Behaviour, Centre for Neuroimaging, Radboud University Nijmegen, Nijmegen, The Netherlands; 3 Donders Institute, Centre for neuroimaging, Kapittelweg 29, P.O. Box 9101, NL-6500 HB Nijmegen, The Netherlands; 4 Department of Internal Medicine, Radboud University Medical Center, Nijmegen, The Netherlands; 5 Department of Psychiatry, Radboud University Medical Centre, Nijmegen, The Netherlands; 6 Behavioral Science Institute (BSI), Radboud University Nijmegen, Nijmegen, The Netherlands; 7 Department of Psychology, University of Marburg, Marburg, Germany

**Keywords:** Chronic fatigue syndrome, Cognitive behavioral therapy, MRI

## Abstract

**Background:**

Chronic fatigue syndrome (CFS) is characterized by profound and disabling fatigue with no known somatic explanation. Cognitive behavioral therapy (CBT) has proven to be a successful intervention leading to a reduction in fatigue and disability. Based on previous neuroimaging findings, it has been suggested that central neural mechanisms may underlie CFS symptoms and play a role in the change brought on by CBT. In this randomized controlled trial we aim to further investigate the neural mechanisms that underlie fatigue in CFS and their change by CBT.

**Methods/Design:**

We will conduct a randomized controlled trial in which we collect anatomical and functional magnetic resonance imaging (MRI) measures from female CFS patients before and after CBT (N = 60) or waiting list (N = 30) and compare these with measures from age and education matched healthy controls (N = 30). By including a large treatment group we will also be able to compare patients that benefit from CBT with those that do not. In addition, to further investigate the role of endocrine and immune biomarkers in CFS, we will determine cortisol and cytokine concentrations in blood, hair and/or saliva.

**Discussion:**

This project creates an unique opportunity to enhance our understanding of CFS symptoms and its change by CBT in terms of neuroanatomical, neurofunctional, endocrinological and immunological mechanisms and can help to further improve future treatments strategies.

**Trial registration:**

Dutch Trial Register #15852. Registered 9 December 2013 (http://www.trialregister.nl/trialreg/admin/rctview.asp?TC=4311)

## Background

Chronic fatigue syndrome (CFS) is characterized by severe fatigue persisting at least 6 months and leading to considerable impairment in daily functioning ([1]; Reeves et al., [2]). Various accompanying symptoms may be present, such as joint and muscle pain, headaches, impaired memory and concentration, and exercise intolerance. The aetiology of CFS is currently unknown; symptoms are not explained by a known medical condition and are not alleviated by rest (Prins et al., [3]). Cognitive behavioral therapy (CBT) for CFS, a treatment that aims to change behavior and cognitions thought to perpetuate symptoms, has shown to be an effective treatment for CFS patients [4]. Following treatment, the majority of CFS patients show improvement in symptoms and a subgroup even fully recovers from CFS (Knoop et al., [5]; White et al., [6]).

Little is known about the mechanism of change of CBT for CFS. Thus far, mediation analysis of randomized controlled trials testing the efficacy of CBT suggested that the reduction of fatigue and disability is mediated by a change in cognitions, such as decreased focusing on symptoms and changes in illness beliefs ([7]; Knoop et al., [8]; Wiborg et al., [9]; Wiborg et al., [10]), rather than changes in physical fitness ([7]; Wiborg et al., [11]). These findings implicate that neural mechanisms underlying these cognitions may play an important role in CFS. This suggestion is further supported by accumulating evidence from neuroimaging studies demonstrating neural abnormalities in CFS patients [12]. Thus, several neuro imaging studies have found reduced grey matter volumes ([13, 14]; Okada et al., [15]), white matter changes (Zeineh et al., [16]). and functional abnormalities in prefrontal, parietal, and limbic regions for CFS patients versus healthy controls ([17–20]; Lange et al., [21]). In addition, a recent positron emision tomography (PET) study demonstrated increases in microglia activity, suggestive for neuroinflammation (Nakatomi et al., [22]). Moreover, one neuroimaging study also demonstrated that treatment of CFS with CBT increased grey matter volume in CFS patients. These changes were located in the dorsolateral prefrontal cortex (DLPFC) [14], a region associated with cognitive-regulatory functions. Similarly, CBT-related anatomical and functional changes have been reported in other conditions such as chronic pain (Seminowicz et al., [23]) and mood and anxiety disorders (Beauregard, [24, 25]; Strauman et al., [23]). Accordingly, we suggest that a reduction in fatigue and disability brought on by CBT for CFS is accompanied and possibly mediated by anatomical and functional changes in the brain.

So far, definitive evidence for this argument is limited because the few existing studies have often used small samples sizes and lacked a patient control group. The inclusion of a patient control condition is necessary to attribute changes seen in the treatment group to the intervention, rather than to time per se or to changes occurring irrespective of the treatment. In this study we will address these issues and aim to further investigate neural mechanisms underlying CFS and its change by CBT using anatomical and functional magnetic resonance imaging (MRI) in a large-sample randomized controlled trial (RCT). In this RCT, we will compare changes between CFS patients that had CBT (N = 60) with patients that were allocated to a waiting period (N = 30). By including the large treatment group we will also be able to investigate the relationship between neural changes and treatment response. Patients will be compared with healthy controls (N = 30) who are also tested twice, separated by 6 months, to control for test-retest effects.

This study will investigate neural mechanisms underlying CFS symptoms and its changes after CBT. The aims are threefold. First, we aim to replicate previous found anatomical CBT-related changes in grey matter volumes [14] using anatomical MRI and qualify these findings by exploring changes in metabolite concentrations and white matter integrity using magnetic resonance spectroscopy (MRS) and diffusion tensor imaging (DTI). Second, we aim to explore the mechanisms of change of CBT for CFS using functional MRI (fMRI) and behavioral testing. Finally, we aim to investigate immune and endocrine functioning in CFS and its change after CBT by assessing various biomarkers in blood, saliva and hair. These three aims are discussed in more detail below.

### Anatomical changes in CFS

Our first aim is to replicate and extend previous found anatomical changes in grey matter volumes [14] by including a larger treatment group and a patient control group. Furthermore, we aim to provide additional information about the nature of anatomical changes by exploring changes in metabolite concentrations using magnetic resonance spectroscopy (MRS) and white matter integrity using diffusion tensor imaging (DTI).

MRS allows for the identification and quantification of various metabolites in the brain like N-acetylaspartate (NAA), myo-inositol, choline, creatine glutamate/glutamine. These metabolite concentrations can inform us about the nature of grey matter changes. For example, while NAA is mainly present in the neurons and provides a marker of neuronal health (Moffett et al., [26]), myo-inositol is mainly associated with glia-activation and provides a marker of neuroinflammation [27]. Previous work in small samples of CFS patients (N < 11) has reported reduced NAA levels in the hippocampus [28] as well as increased choline/creatine ratios in the occipital cortex (Puri et al., [29]), prefrontal cortex (Tomoda et al., [30]) and basal ganglia (Chaudhuri and Behan, [31]) , compared with healthy controls. Similarly, alterations in MRS measures have been reported in other medically unexplained conditions such as chronic pain (see review (Harris and Clauw, [32])) and irritable bowel syndrome (Niddam et al., [33]). MRS measures have shown to vary as a function of pain sensitivity ([34]; Petrou et al., [35]) or pain catastrophizing ([36]; Niddam et al., [33]). Moreover, some pilot studies show that NAA levels are subject to change following behavioral therapies [37] (O’Neill et al., [38]; Zurowski et al., [39]) suggesting that NAA reductions are reversible and can be used to monitor treatment response. In this project, we will investigate CBT-related metabolite changes in the DLPFC, as our aim is to assess the nature of previously reported grey matter changes in this region [14]. However, given that previous studies in CFS found metabolite differences in various brain regions, regional specificity to the DLPFC will be investigated by comparing changes in the DLPFC with those in the occipital cortex.

DTI is a technique that is sensitive to microstructural organization of neural tissues, measuring both the directionality and the magnitude of water diffusion (Beaulieu, [40]). To our knowledge, only one study has used DTI to investigate white matter in a small sample of CFS patients (N = 15) and report increased fractional anisotropy (FA) values in CFS patients compared with healthy controls in the right arcuate fasciculus, a white matter pathway that connects the frontal lobe with the inferior parietal lobe (Zeineh et al., [16]). In this project we will assess DTI measures in a larger sample and also explore possible changes after CBT.

### Functional changes in CFS

Our second aim is to explore the mechanisms of change of CBT for CFS by means of functional MRI and behavior. Task-dependent functional neuroimaging provides information about neural mechanisms that underlie specific processes involved in the execution of a specific task. Accordingly, task selection should always be done against the background of relevant cognitive and neural models of the disorder ([12]; Strauman et al., [23]). For this project, task-selection was based on a combination of a neurobiologically informed Bayesian model of somatoform symptoms [41] and clinical observations. The recently developed Bayesian model describes somatoform symptoms in terms of altered perception resulting from an inference failure between sensory information and prior beliefs [41]. In the light of CFS, the model suggests that increased perception of symptoms may arise from pathologically precise prior beliefs that affect sensory perceptions and attributions. This model exploits some key factors that have been identified by clinical cognitive behavioral models of CFS and that are challenged by CBT for CFS including the role of fatigue related beliefs (e.g. about the ability to engage in physical activities) and the attention towards symptoms ([7]; Knoop et al., [8]; Vercoulen et al., [42]; Wiborg et al., [9]). In addition, clinical observations demonstrate that symptoms often involve physical activities. Thus, CFS patients show reduced activity levels (Nijs et al., [43]; Werf et al., [44]) and underperformance on physical exercise tasks (Fry and Martin, [45]; Riley et al., [46]; Schillings et al., [47]). These decreases in physical activities are suggested to result from reduced expectations about physical abilities (Heins et al., [48]; Nijs et al., [49]), fear avoidance beliefs (e.g. the fear that exercise will make symptoms worse) and avoidance behaviors, which are highly prevalent in the CFS population ([50]; Nijs et al., [51]). Indeed, a recent clinical trial revealed that changes in fear avoidance beliefs and avoidance behavior mediate the CBT effects in CFS patients [7]. Following the neurobiological informed model and these clinical observations, we aim to investigate neural mechanisms underlying inference processes during effortful physical exertions using two fMRI paradigms. In addition, we will also explore general inference processing in the visual domain using a behavioral paradigm.

In the first fMRI paradigm we will investigate effort perception during an effort task, using a MR-compatible handgrip device. Neural activity will be assessed during preparation of low, medium and high effort production and in response to task-feedback indicating that the subjects provided too much, too little or the correct amount of effort. Previous work has already demonstrated that CFS patients show alterations in neural error processing during a motor imagery task [20]. This study will extend these findings by testing whether alterations in motor preparation and error processing are consistent with the known fatigue-related beliefs. To this end, we will test the hypothesis that CFS patients show alterations in neural feedback-processing towards errors that indicate that too little versus too much physical effort has been produced and that these alterations vary as a function of effort level. We hypothesize that successful CBT outcome is accompanied by normalization of these alterations.

In the second fMRI task we aim to investigate alterations in neural processes underlying effort avoidance learning. CFS has been associated with increased avoidance of physical activities (see above). However, it is unclear whether these avoidance behaviors are due to the increased experienced effort of physical activities or whether CFS patients develop dysfunctional beliefs about the need to avoid physical activities. We will assess neural mechanisms underlying the inferring of contingencies between cues and effortful handgrip-contractions, using an adapted version of the salience attribution task (Roiser et al., [52]; Roiser et al., [53]). This task allows for dissociating avoidance learning that is adaptive, i.e. developing avoidance only for cues that are predictive for effortful outcomes, from avoidance learning that is non-adaptive, i.e. developing dysfunctional avoidance, also for cues that are not predictive for effortful outcomes. We will test two hypotheses: 1) CFS patients will show increased adaptive avoidance learning compared to healthy controls, which is suggestive of an increased impact of the experienced effort on avoidance learning. 2) CFS patients will show increased non-adaptive avoidance learning, which is suggestive of a higher tendency to develop dysfunctional expectations for cues that are not predictive for effortful outcomes. We hypothesize that successful CBT is accompanied by normalization of (non-) adaptive avoidance learning.

In the behavioral task we aim to investigate general inference processes in CFS patients. The Bayesian model suggests that the perception of medically unexplained symptoms result from prior beliefs that are afforded too much precision. Thus, an increased general tendency to base perceptual decisions more on prior information than on sensory inputs in any sensory domain may reflect a fundamental vulnerability that may predispose subjects to develop CFS. Here we aim to test this hypothesis in the visual domain by investigating the influence of informative cues on decisions about the direction of moving dots using the well developed moving dot paradigm (Rahnev et al., [54]).

### Immune and endocrine changes in CFS

Finally, we aim to investigate immune and endocrine changes in CFS. Cytokines are small proteins that play a key role in normal physiology and disease. Because of the resemblance of CFS symptoms with so called “sickness behavior” including the symptoms fatigue, post-exertional malaise and reduced activity levels, CFS has often been suggested to involve increased activity of pro-inflammatory cytokines, such as IL-1β and TNF-α which are considered to play a key role in inducing sickness behavior during acute illness ([55]; Kelley et al., [56]). In this project we will not only assess cytokine concentrations in blood plasma, but also evaluate cytokine activity at the mRNA level using pax-gene tubes. Previous studies on this subject have met with inconsistent results (Lyall et al., [57]). One explanation is that cytokines in the body respond to a variety of external and environmental stimuli like stress and exercise. To control for such influences, we will select well-matched controls that will follow exactly the same procedures during each test day. In addition, cytokines will not only be measured in blood plasma, but also at the mRNA level using pax-gene tubes.

Disturbances in hypothalamic-pituitary-adrenal (HPA)-axis functioning have been associated with fatigue and CFS symptoms (Powell et al., [58]; Silverman et al., [59]; Tak et al., [60]). More specifically, previous work shows that CFS patients are characterized by hypo-cortisolism (Nater et al., [61]a; Nater et al., [62]b), which can normalize to levels comparable to healthy population after CBT in adolescents (Nijhof et al., [63]; Rimes et al., [64]) and adults (Roberts et al., [65]). In this project we will replicate and extend these findings, by assessing both diurnal cortisol fluctuations in saliva as well as baseline cortisol levels of the past month in hair.

### Summary

This project aims at investigating neural mechanisms underlying CFS symptoms and its changes after CBT by collection anatomical MRI measures, functional MRI measures, behavioral measures and blood, saliva and hair sampling. We will extend previous neuroimaging work by collecting data form a large group of subjects and by including a patient control condition in a RCT. This project creates a unique opportunity to enhance our understanding of CFS symptoms and its change by CBT in terms of neuroanatomical, neurofunctional, behavioural, immunological and endocrinological mechanisms.

## Methods/design

### Study design

**Fig. 1:**
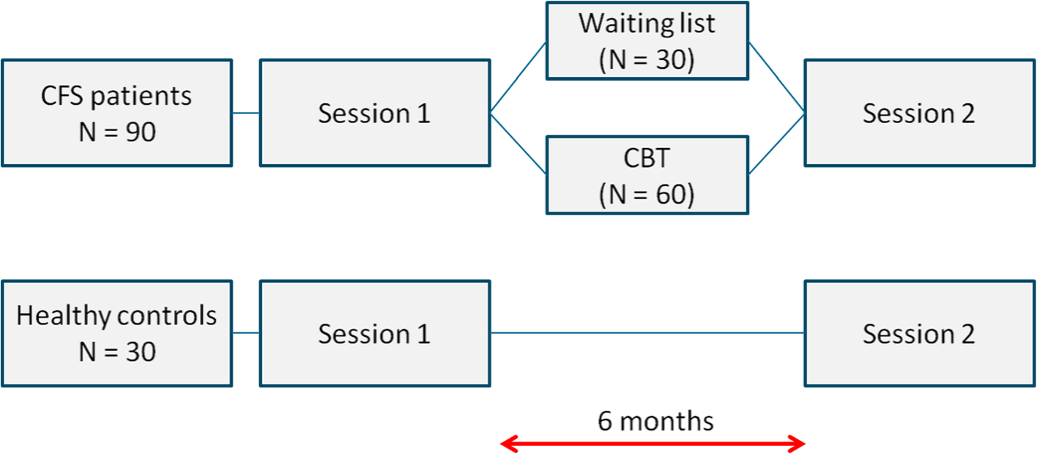
Trial design. CBT = Cognitive behavioural therapy

A RCT will be conducted to investigate neural correlates of change brought on by CBT in patients diagnosed with CFS. CFS patients will be randomly assigned to either the intervention group (N = 60), which will immediately start with CBT, or the waiting group (N = 30), which will start with CBT after a 6 months waiting period. In order to compare CFS patients that recover from CBT with those that do not we will use a 2:1 allocation ratio for CBT and waiting group respectively. MRI measurements, behavioral measures, questionnaires and biomarker sampling will be gathered at baseline and at a second session after approximately 6 months in both groups. Results will be compared to 30 age, gender and education matched healthy controls, which will also be tested twice, separated by a 6 months period (Fig. 1).

### Ethical aspects

The study is approved by the Medical research ethics committee and Central Committee on Research Involving Human Subjects (registration number NL43606.091.13). This study will be conducted according to the principles of the declaration of Helsinki. CFS patients will be recruited from the Expert Centre for Chronic Fatigue (ECCF). Participants will be informed about the study and written informed consents will be obtained before the first testing day and randomization.

Participants can withdraw from the study at any time, and for any reason, without consequences. The investigator can decide to withdraw a participant from the study in case of late disclosure of exclusion criteria (e.g. psychiatric co-morbidity) or a change in meeting in- and exclusion criteria (e.g. when participant decides to start psychotropic medication after inclusion). CFS patients who (are) withdraw(n) from the study will continue to receive treatment at the ECCF. Patients that withdraw before randomization or did not start face-to-face CBT when assigned to the CBT condition and participants that are withdrawn by the investigator will be replaced when time allows. Patients that received CBT for CFS outside the ECCF during their waiting list period will be excluded from analysis.

### Study population and recruitment procedure

A total of 90 female CFS patients and 30 age and education matched female healthy controls will be recruited from the ECCF of the radboudUMC in Nijmegen, the Netherlands. Only female patients are included for homogeneity. Patients that are referred to the ECCF of the Radboudumc, are diagnosed with CFS according to the US Centers for Disease Control and prevention (CDC) consensus-criteria ([66]; Reeves et al., [2]), and scoring 40 or higher on the subscale fatigue severity of the Checklist Individual Strength (CIS) and have a total score of 700 or higher on the Sickness Impact Profile 8 (SIP8) will be informed about the study by their treating psychologist during their first intake session at the ECCF. If the patients agree, they will receive an information folder. The investigator will call the patients after a one week reflection period to answer any further questions and to ask for participation. If willing to participate, and meeting all in- and exclusion criteria (Table 1), patients will be invited for the first testing day at the Donders institute for brain cognition and behavior. Age and education matched female healthy controls will be recruited though advertisements and through flyers in the patients folder. Interested healthy controls will receive an information folder and the recruitment procedure will follow the same steps as described above. Written informed consent will be given at the first session day. Randomization of patients will take place after the first session day. The Mini-international Neuropsychiatric Interview (M.I.N.I) will be used to rule out any current psychiatric disorders.

**Table 1:** Inclusion and exclusion criteria

Inclusion criteria	
1.	Female
2.	Age between 18 and 55 years
Additional inclusion criteria for CFS patients	
1.	CFS diagnosis according to the CDC-criteria ([66]; Reeves et al., [2]);
2.	Fatigue severity subscale (CIS) score ≥40
3.	Severely disabled, defined by scoring ≥ 700 on the Sickness Impact Profile (SIP)
4.	Eligible for treatment with CBT
Additional inclusion criteria for healthy controls	
5.	Fatigue severity subscale (CIS) score ≤ 35
Exclusion criteria	
1.	Current use of psychotropic medication (e.g. antidepressants)
2.	Contra-indication for MR-examinations (e.g. claustrophobia)
3.	Abnormal hearing or (uncorrected) vision
4.	Insufficient command of the Dutch language to fill out questionnaires, understand task instructions or perform the neuropsychological tests
5.	Restricted function of the right hand that confounds handgrip performance for the fMRI tasks
Additional exclusion criteria for healthy controls	
6.	Current psychological or psychiatric disorder^a,b^
7.	(History of) alcohol or substance abuse^a^
8.	Severe obesity (BMI ≥ 40)^a^
9.	Chronic disease or pain condition (e.g. rheumatoid arthritis)
10.	Current acute pain condition

^a^these criteria are part of the CDC criteria for CFS

^b^as revealed by the Mini International Neuropsychiatric Interview (M.I.N.I.)

### Intervention

CBT for CFS (Heins et al., [67]; Wiborg et al., [9]) aims at recovery, meaning that the CFS patient is no longer severely fatigued and disabled by the fatigue, by changing cognitions and behaviors assumed to perpetuate the symptoms. Previous trials have repeatedly demonstrated that CBT for CFS leads to a significant reduction of fatigue and disability ([68]; Prins et al., [69]; White et al., [6]; White et al., [70]; Wiborg et al., [9]) and to recovery in a subset of patients (Knoop et al., [5]; White et al., [6]). CBT treatment will be given by trained cognitive behavioral therapists at the ECCF of the radboudUMC in Nijmegen, the Netherlands. Treatment consists of 12–14 individual sessions with a trained cognitive behavioral therapist within a period of approximately 6 months. The treatment starts with explanation of the cognitive behavioral model of CFS and goal setting. Patients formulate their goals in concrete activities they will do when they are no longer bothered by fatigue, e.g. resumption of work or study. Then, patients will learn to regulate their sleep-wake cycle by maintaining fixed bedtimes and no longer sleep or lie down during the day. Once the sleep-wake cycle is regulated, patients will start with a graded activity program consisting of a graded increase in walking of cycling activities. Based on their physical activity patterns, relative active patients will first learn to better divide activities across the day before starting with the graded activity program and low active patients will start directly with the graded activity program. Dysfunctional fatigue-related cognitions, e.g. catastrophising in response to fatigue, low self-efficacy and increased focus on symptoms are challenged continuously throughout the intervention. When activity levels are increased and patients attain the belief that they have potential to be more active, they start with realization of their prior set goals. The last part of the intervention aims at reappraisal of fatigue as a normal sensation and prevention of relapse.

### Outcome measures

#### Primary clinical outcome measures

Fatigue severity will be measured using the subscale fatigue severity of the CIS consisting of 8 items scored on a 7-point Likert Scale with a total score ranging between 8–56 (Vercoulen et al., [71]). Higher scores indicate higher levels of fatigue. This questionnaire has been validated extensively in patients with CFS ([72, 73]; Vercoulen et al., [71]; Vercoulen et al., [42]). Following previous reports (Prins et al., [69]; Wiborg et al., [10]) we will use cut-off score of ≥ 40 for inclusion of severely fatigued CFS patients.

Successful treatment is defined by a clinically significant improvement on the CIS sub-scale fatigue severity, i.e. scoring lower than 35 and a reliable change index of >1.96 on the CIS subscale fatigue (Knoop et al., [74]). The cut-off score of 35 has frequently been used as a criterion to indicate clinically relevant fatigue in clinical studies (Knoop et al., [5]; Wiborg et al., [75]).

Functional impairment will be measured in CFS patients using the Sickness Impact Profile 8 [76]. The SIP8 contains 85 statements about health-related dysfunction in 8 areas domains (home management, ambulation, mobility, intellect, social interactions, sleep/rest behaviour, recreation and work). In completing the SIP, the patient is asked to check only those statements that she is sure describe her on a given day and are related to her health. The eight subscales are added to provide one weighted score of disability (SIP8 total, range 0–5799) (Knoop et al., [5]; Prins et al., [69]; Wiborg et al., [9]). Higher scores indicate higher levels of experienced disabilities. Functional impairment will be defined as a SIP score higher than 700.

#### Secondary clinical measures

Fatigue related cognitions and behaviours, physical and social functioning are assessed using questionnaires as part of the clinical routine of the ECCF. A selection of these questionnaires will also be assed in the healthy control group and include the RAND-36 (Stewart et al., [77]; Ware and Sherbourne, [78]), the Beck depression Inventory (BDI) [79], the Symptom Checklist 90 (SCL-90) (Arrindell and Ettema, [80]), the Fatigue Quality List (KWAMOE) [81], the self efficacy scale fatigue (SES) (Prins et al., [69]; Vercoulen et al., [71]; Wiborg et al., [9]) and the Adjusted Physical Activities Rating Scale (PARS) (Vercoulen et al., [82]). In addition, the attention Control Scale questionnaire (ACS)(Derryberry and Reed, [83]) will be assessed in all subjects. During each test day, state changes in fatigue and mood will be assessed using the profile of moods (POMS) questionnaires (McNair et al., [84]) at 3 time points in both patients and healthy controls.

### Physical activity

Previous work in both humans[85] and animals (Praag, [86]) suggest a possible link between physical exercise and neurogenesis. Accordingly, given that graded increase of physical activity levels is an important part of the CBT program, we will also assess physical activity levels. As in the previous reports [13, 14], physical activity levels will be assessed in all participants, over a period of two weeks preceding both test sessions using an actometer. The actometer is a motion-sensitive device, worn at the ankle, which can register and quantify human physical activity (Werf et al., [44]).

### Neuropsychological testing

To be able to replicate previous reported correlations between grey matter volume changes and neuropsychological performance [14] we will assess two tasks of cognitive speed: the digit symbol substitution test of the Dutch Wechsler Adult Intelligence Scale (Stinnissen J et al., [87]) and a choice reaction time task ([14]; Vercoulen et al., [42]). Additionally, working memory capacity will be assessed with the digit span of the Dutch Wechsler Adult Intelligence Scale (Groth-Marnat, [88]; Stinnissen J et al., [87]) and intelligence/education level will be determined with the Dutch adult reading test (Schmand et al., [89]).

### Structural neuroimaging measures

To investigate changes in brain anatomy and metabolism, several MR-measurements will be obtained on a 3-Tesla SIEMENS MAGNETON skyra MRI scanner. To investigate global and regional grey and white matter changes high resolution anatomical images will be obtained using a T1 weighted magnetization-prepared rapid gradient-echo (MP-RAGE) sequence (TR\TE: 2300\3.03 ms, flip angle = 8°, 192 sagittal slices, FoV: 256 × 256 mm, voxelsize: 1 mm^3^, slice thickness: 1.00 mm). To investigate the microstructural properties of white matter pathways in the brain, diffusion tensor imaging (DTI) scans will be assessed (TR = 8200 ms, TE = 89.0 ms, voxel size: 2.2 × 2.2 × 2.2 mm, FOV 220 mm, 64 slices, slice thickness: 2.2 mm). Brain metabolite changes will be assessed using single voxel proton magnetic resonance spectroscopic (MRS) imaging (TR = 1500 ms, TE = 30 ms, and 64 averages). MRS spectra will be obtained from two voxels (10 × 20 × 30 mm): One voxel placed in the left middle frontal gyrus, corresponding to the previous reported region of CBT-associated grey matter changes in CFS patients [14] and one voxel placed in the left calcarine gyrus (i.e.primary visual cortex) to assess regional specificity of the changes.

### Functional MRI

To explore task-dependent functional MRI will be assessed using a multi echo, T2*-weighted, gradient-echo planar imaging (EPI) sequence (TR = 2190 ms, TE = 9.0/19.28/29.56/39.84 ms, flip angle = 90°, 36 axial slices aligned with AC-PC plane, slice-matrix size = 64 × 64, slice thickness = 3.0 mm, slice gap = 0.3 mm, FOV = 212*212 mm). Two different tasks will be employed.

With the first task, we will assess alterations in brain activation patterns in anticipation of effortful physical exertions and in response to task-feedback about these exertions. Participants are instructed to prepare and then squeeze quickly in a hand grip up to a certain level indicated by a cue. Cues will indicate to squeeze 30, 50 or 70 % of their maximal voluntary contraction (MVC). MVC will be calibrated before and after the task to assess (change in) physical ability. Subjective experience of fatigue and effort will be assessed before and after the task. Visual feedback is provided after the squeeze and will indicate whether the squeeze was “correct”, “too little” or “too much”. Neural outcome measure is the Blood Oxygen Level Dependent (BOLD) contrast during task performance. Behavioral output is the squeezed force. We will assess the hypothesis that, compared with healthy controls, CFS patients will show alterations in neural response to “too much” and “too little” and that these alterations will vary as a function of effort level. We expect that these alterations will normalize after successful treatment. In addition, neural responses during effort anticipation will be explored.

With the second task, we will assess alterations in neural activation patterns during effort avoidance learning using an adapted version of the salience attribution task (Roiser et al., [52]; Roiser et al., [53]). In this task participants learn to reduce the effort level of handgrip contractions by responding sufficiently quickly to a target presented after a cue. The cues have two dimensions (shape and color) that provide information about the probability of the occurrence of the effortful handgrip contractions. One dimension is predictive for the outcome, signaling the occurrence of the effortful handgrip contractions with 80 % and 20 % certainty. The other dimension is non-predictive for the outcome, signaling the occurrence of the effortful handgrip contractions with 50 % certainty for both cues. When effortful outcomes occur, the required effort level of the handgrip contraction is reduced as a function of reaction time to the target. Thus, the faster the subject responds to the target, the lower the effort level. Neural outcome measure is the Blood Oxygen Level Dependent (BOLD) contrast during task performance. Explicit and implicit avoidance learning will be assessed using cue ratings assessed after the task, and response reaction times, respectively. Adaptive avoidance learning is defined by higher neural responses, faster reaction times and higher explicit ratings for high relative to low predictive cues. Non-adaptive avoidance learning is defined by larger differential neural responses, reaction times and explicit ratings for the two non-predictive cue dimensions. We will assess whether CFS patients will show increased adaptive and/or non-adaptive avoidance learning compared to healthy controls and whether this normalizes after CBT.

Task independent-neuroimaging will be used to explore alterations in neural networks during rest that may underlie CFS symptoms and CBT effects. A 5 minute resting state scan will be obtained to explore task-independent functional connectivity patterns using a multi echo T2*-weighted, gradient-echo planar imaging (EPI) sequence (TR = 2000 ms, TE = 9.0 ms, flip angle = 90°, voxel size = 3.3 × 3.3 × 3.3 mm, slice thickness: 3.0 mm). During the scan, the room will be completely dark and subjects are asked to lie still with their eyes open to avoid falling asleep.

### Behavioral task

We will use a behavioral version of the moving dot paradigm ([90]; Rahnev et al., [54]) to investigate the influence of expectancy cues on perceptual decisions. Subjects are presented with motion of white dots and are requested to indicate the direction of movement with a button press (i.e. left or right). Moving dots are presented using three coherence levels, determined by subjects’ accuracy level during a preceding training session. Prior to each stimulus subjects are presented with the words “LEFT” or “RIGHT” indicating the direction of motion with 75 % accuracy or with the non-informative word “NEUTRAL”. After each trial feedback is given by changing the colour of the fixation point to green (correct) or red (incorrect). Behavioural outcome measures are the direction of the movement indicated by the participant and reaction times. As described by ([90]; Rahnev et al., [54]), subjects’ hit rates will be used to calculate individual stimulus discrimination sensitivity (d’) and bias towards the cued direction (c). Following Edwards model (see introduction) we will test the hypothesis that CFS patients show a larger bias towards the cued direction compared to healthy controls and whether this changes following CBT.

### Citokines and cortisol

Circulating cytokine concentrations will be determined in blood-plasma using a commercial multiplex assay. Using pax-gene tubes, cytokine mRNA concentrations will be measured. The latter will give more insight into upstream regulation of local cytokine production. Cortisol concentrations will be determined from saliva and hair. We will measure both daily variations of cortisol levels (i.e. cortisol awakening response) in saliva as well as baseline cortisol levels of the past month in hair. Blood and hair samples will be assessed on each test day. Saliva will be collected at home during two consecutive weekdays within two weeks prior to or after each test day. Saliva samples will be taken at 4 different time points during a day: directly after waking, 30 minutes after waking, around 11.00 a.m. or at least 1 hour after the second sampling and around 08.00 p.m. Cytokine and cortisol concentrations will be compared between CFS and HC and before and after CBT or waiting list.

### Session procedure

**Fig. 2:**
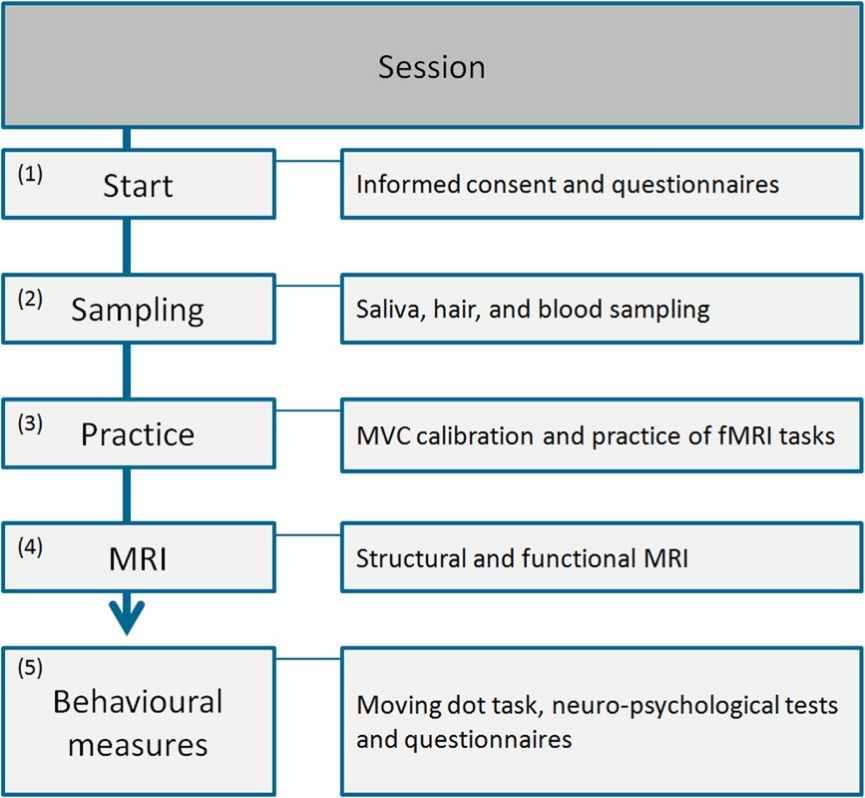
Flowchart of fMRI session day. MVC = Maximal voluntaty contraction, MRI = Magnetic resonance imaging, fMRI = functional MRI

Participants will be invited to the Donders institute for brain cognition and behavior on two session days separated by approximately 6 months. Each session will include the following procedures: (1) hair and blood sampling. (2) practice of fMRI tasks, including the calibration of MVC for the two fMRI tasks. The practice session is done in a dummy scanner to ensure same body position during handgrip-calibration, practice and fMRI-scanning. (3) MRI scanning, including six scans and a 15 minute break: structural MRI, fMRI during task1, MRS, break, fMRI during task2, DTI and resting state. (4) Behavioral and neuropsychological testing. The order is kept the same across subjects and sessions. An overview of a session day is given in Fig. 2. Written informed consent is given on the first session day. Trait measures such as the adult reading test are only assessed on the first test day. POMS measurements are assessed before sampling, during the break and after behavioral testing. The total duration of each session will be about 5 hours. For CFS patients, questionnaires are assessed as part of the clinical routine of the ECCF prior to the session days. For healthy controls, questionnaires are assessed at home through an internet link, sent to the subjects by email.

### Analysis

The aim of this project is to explore the neural mechanisms of change underlying (successful) CBT for CFS. Accordingly, we will include all patients that completed both sessions for analysis of CBT effects. Statistical analysis will be done to 1) compare outcome measures between CFS patients and healthy controls at baseline, 2) compare changes between baseline and second assessment between patients that had CBT and waiting list and 3) compare changes between baseline and second assessment between CFS patients within the CBT condition that show clinical significant improvement (see above) and those that do not. Following previous reports [13, 14], relationship between neural outcome measures and clinical outcome measures, physical activity levels and neuropsychological functioning will be assessed. Multiple mediation analyses will be conducted to explore whether neural outcome measures mediate therapy effects on clinical outcome measures (Preacher and Hayes, [91]).

### Sample size

**Fig. 3:**
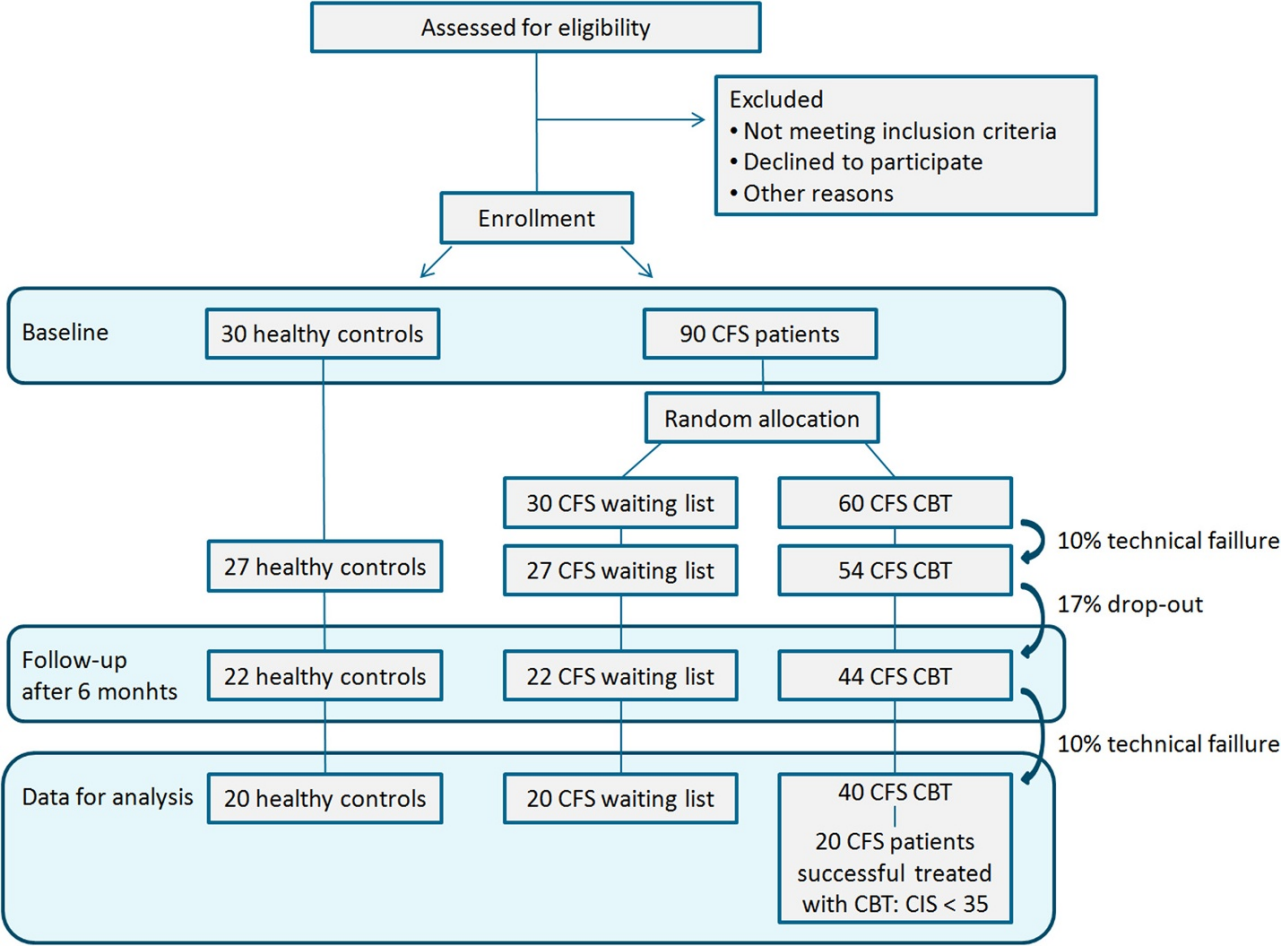
Flowchart of inclusion. CFS = Chronic fatigue syndrome, CBT = Cognitive behavioural therapy

Power calculation is based on current standards in neuroimaging. For (f)MRI, the recommended number of subjects for between-group designs is 20 (Thirion et al., [92]). In addition, from previous studies we know that groups of 20 subjects per condition are sufficient to detect significant differences in (neuro) physiological parameters measured with fMRI [17, 18]. Similarly, a group of 22 subjects were sufficient to detect significant treatment effects measured with MRI [13]. Based on previous work at the ECCF (Knoop et al., [5]) it is assumed that approximately 50 % of the CFS patients in the CBT condition will show significant improvement (for definition: see above). Accordingly, to be able to compare successfully treated patients with non-successfully treated patients we will include twice as many patients in the CBT condition as in the waiting list condition. Assuming that 10 % of the measurements (from baseline as well as 2nd assessment) will yield technically insufficient data, 17 % of the patients will drop out from the study and a ratio of 2:1 (CBT: waiting list), we will include 30 healthy controls, 30 patients in the waiting list condition and 60 patients in the CBT condition (Fig. 3). Based on previous clinical work (Knoop et al., [74]; Wiborg et al., [9]), we expect a mean decline of–15.2 (±15.8) for patients in the CBT condition and–5.2 (±7.2) for patients in the waiting list condition. Accordingly, assuming an alfa of 0.05 and a power of 0.95 this will also yield enough power to replicate previous reported treatment effects on the primary clinical outcome measures. Note that technical failure of MRI measurements will not affect the collection of clinical measures.

### Randomization

CFS patients will be randomized to either the CBT condition (66 %) or waiting list condition (33 %). Randomization will take place per patient after the first session day in the presence of the therapist and patient, either during the second diagnostic appointment with the therapist or during a phone call with the therapist. Randomization will be done by supporting staff of the ECCF who are not directly involved in the study. Concealed allocation will be guaranteed by using a central web-based randomization program, developed by an independent statistical advisor. Patients randomized to the intervention group will start treatment directly and will be treated with CBT for CFS (see above). Patients randomized to the control group will start with CBT for CFS 6 months later, after the second assessment. The researchers that will collect and analyze the data are not blind to the allocation, because knowledge of treatment progress is necessary for the planning of the second test day.

## Discussion

This study will investigate neural mechanisms underlying CFS symptoms and its changes after CBT. The aims are threefold. First, we will replicate and extend previously found anatomical findings, second, we will explore underlying neural mechanisms using functional neuroimaging and third, we will collect biomarker measures from blood, hair and saliva to assess cytokine and cortisol changes during CBT.

To our knowledge, this will be the first randomised controlled trial investigating neural mechanisms of change brought on by CBT for CFS. There are a limited number of studies that investigated neural changes after CBT in CFS [14] or CBT in other disorders (Seminowicz et al., [93]). However, those studies often relied on small samples (<20) and seldom used a no-treatment patient control condition. Here we overcome those problems by collecting data of more patients and by including a waiting list control condition. Because we will include twice as many patients in the CBT condition as in the waiting list condition, this study will allow us not only to investigate changes between CBT and waiting list conditions, but also to investigate changes within the CBT group between those patients that benefit from CBT and those that do not. This creates a unique change to investigate the neural mechanisms of change of CBT and to identify biomarkers that may predict treatment outcome.

We aim to replicated previously found anatomical findings [13, 14] and extend these findings with additional anatomical information using MRS and DTI. With respect to functional neuroimaging and behavioral assessment, task selection and hypotheses are based on a combination of clinical observations, cognitive behavioral models of CBT and a recent neurobiologically informed Bayesian model of medically unexplained symptoms. This translation between clinical observations and fundamental neuroscience models is unique in the investigation of CFS and an important step forward in understanding the etiology of CFS symptoms and the mechanisms of change of CBT.

Taken together, this project creates a unique opportunity to enhance our understanding of CFS symptoms and its change by CBT in terms of neuroanatomical, neurofunctional, behavioural, immunological and endocrinological mechanisms. Moreover, the comparison of patients that benefit from CBT with those that do not may lead to new insights for treatment resistant CFS patients.
